# 43.3 COMPLEMENT DYSREGULATION IN SCHIZOPHRENIA: IMPLICATIONS FOR POTENTIAL TREATMENT STRATEGIES

**DOI:** 10.1093/schbul/sby014.181

**Published:** 2018-04-01

**Authors:** Clare Beasley

**Affiliations:** University of British Columbia

## Abstract

**Background:**

Dysregulation of the immune system and inflammation likely play a role in the development or course of schizophrenia, at least in a sub-population of patients. In particular, recent evidence has implicated the complement system in this disorder. The complement system is a key effector of innate immunity, mediating elimination of pathogens and debris via initiation of phagocytosis, inflammation and cell lysis. Intriguingly, early complement components also participate in synaptic pruning and plasticity. Further evaluation of the role of the complement system in schizophrenia may reveal novel treatment strategies.

**Methods:**

Investigations of the complement system in blood and postmortem brain tissue in schizophrenia will be reviewed. Furthermore, the relationship between blood and brain complement levels and measures of central and peripheral inflammation, genotype and synaptic density will be explored. Finally, the potential utility of anti-complement therapies in the treatment of schizophrenia will be discussed.

**Results:**

Recent genome-wide association studies have revealed an association with genetic markers within the major histocompatibility complex locus in schizophrenia, with this association suggested to reflect diversity in complement component 4 (C4) genes. Consistent with genetic data, higher complement hemolytic activity has been reported in this disorder, while our studies in postmortem brain tissue have revealed increased expression of several complement components. Notably, C4 expression is impacted by C4 genetic architecture.

**Discussion:**

Overall, data suggests a role for the complement system in schizophrenia. Increased complement expression may be indicative of an inflammatory response. However, given that early complement components have also been implicated in synaptic pruning and circuit remodeling, disturbances in complement activity may also contribute to synaptic deficits previously identified in this disorder. Anti-complement therapies are currently available, while the complement system provides numerous additional options for future drug development. Further research is required to elucidate the potential utility of anti-inflammatory therapies, including those targeting the complement system, in the treatment of schizophrenia, and to identify which patients may benefit most from this strategy and when treatment would be most effective.

